# Simultaneous assessment of blood flow changes in femoral artery and skeletal muscle microvasculature using pulsed-wave and power Doppler ultrasonography

**DOI:** 10.1007/s00421-025-05813-2

**Published:** 2025-05-14

**Authors:** Kazuma Izumi, Kana Shiozawa, Yutaka Kano, Keisho Katayama, Noriko Tanaka, Hiroshi Akima

**Affiliations:** 1 Graduate School of Education and Human Development, Nagoya University, 1 Furo, Chikusa, Nagoya, Aichi 464-8601 Japan; 2 Graduate School of Medicine, Nagoya University, 65 Tsurumai, Showa, Nagoya, Aichi 466-8550 Japan; 3 Research Fellow of Japan Society for the Promotion of Science, 5-3-1 Kojimachi, Chiyoda, Tokyo, 102-0083 Japan; 4 Department of Engineering Science, Bioscience and Technology Program, University of Electro-Communications, 1-5-1 Chofugaoka, Chofu, Tokyo, 182-8585 Japan; 5 Research Center of Health, Physical Fitness and Sports, Nagoya University, 1 Furo, Chikusa, Nagoya, Aichi 464-8601 Japan

**Keywords:** Femoral artery blood flow, Intramuscular blood flow, Power Doppler ultrasonography, Muscle oxygenation, Exercise

## Abstract

**Purpose:**

The purpose of this study was to evaluate blood flow changes in femoral artery and skeletal muscle microvasculature during intermittent submaximal isometric knee extension.

**Methods:**

Seventeen healthy young males (19.7 ± 1.2 years) performed intermittent (5 s on, 5 s off) isometric knee extension. Five contractions were performed at each force level of 10%, 30%, 50%, and 70% of maximal voluntary contraction (MVC) at random with a 10-min rest between sets. We measured right femoral artery blood flow by pulsed-wave Doppler ultrasonography and intramuscular blood flow in the vastus lateralis of the right mid-thigh by power Doppler ultrasonography, simultaneously. Both femoral artery and intramuscular blood flow were normalized by the peak value for each participant and represented as %Peak. Time-to-peak was defined as the time from the end of exercise to the peak.

**Results:**

%Peak of femoral artery blood flow was significantly higher than that of intramuscular blood flow at the baseline and following contractions at 10% MVC (*P* < 0.01). The time-to-peak during the post-exercise of intramuscular blood flow was significantly longer than that of femoral artery blood flow following contractions at 70% MVC (*P* < 0.01).

**Conclusions:**

These results indicate that blood flow increases appeared slowly in skeletal muscle microvasculature than in femoral artery after intermittent submaximal isometric knee extension, suggesting that differences in vascular reactivity and blood flow regulation could exist between femoral artery and skeletal muscle microvasculature in healthy young males.

## Introduction

Blood flow increases to meet the metabolic demands of the working muscles during exercise (Saltin 1985), and it plays a crucial role in supplying oxygen and eliminating metabolic by-products. Therefore, blood flow impairment strongly influences exercise performance. Previous studies showed that blood flow through the femoral artery, a conduit artery supplying blood to the lower limb, linearly increases with exercise intensity, as measured by pulsed-wave Doppler ultrasonography (Rådegran 1997; Osada et al. 2015). The increase in femoral artery blood flow induced by repetitive knee extension exercises has been interpreted to reflect an increase in blood flow within the quadriceps muscle (Rådegran 1997; Osada et al. 2015). However, conduit arteries supply blood to both contracting skeletal muscles and inactive tissues, such as unrecruited muscles, adipose tissue, the skin, and bone (Heinonen et al. 2014). Therefore, whether femoral artery blood flow solely reflects blood flow changes within the quadriceps muscle is difficult to determine.

Studies have shown that the changes in blood flow during exercise (Harper et al. 2006; Schlup et al. 2015; Hammer et al. 2018) and whole-body vibration as an exercise alternative (Betik et al. 2021) differ between conduit arteries and the skeletal muscle microvasculature. According to Harper et al. (2006), the temporal changes in blood flow of muscle capillaries are considerably slower than that of conduit arteries. Betik et al. (2021) compared microvascular blood flow in the vastus lateralis between intermittent isometric knee extension and whole-body vibration. Microvascular blood flow was significantly higher with knee extension exercises than with whole-body vibration, despite the increase in femoral artery blood flow being comparable between the trials. However, in these previous studies, invasive techniques (Betik et al. 2021) and indirect blood flow estimation (Harper et al. 2006) were used to assess blood flow in the active muscles. Non-invasive and direct approaches are more desirable for intramuscular blood flow measurement.

Recent studies have shown that power Doppler ultrasonography has the potential to assess intramuscular blood flow during exercise (Dori et al. 2016; Heres et al. 2018; Izumi et al. 2025). Power Doppler ultrasonography is non-invasive and has high temporal resolution, and it can be used to determine the volume of blood in the vascular bed (Martinoli et al. 1998). Our recent study demonstrated that intramuscular blood flow within the vastus lateralis reached a plateau above moderate-intensity intermittent incremental isometric knee extension exercise (Izumi et al. 2025). As mentioned above, the changes in femoral artery blood flow may not always reflect changes in intramuscular blood flow. The simultaneous assessment of femoral artery and intramuscular blood flow could help to elucidate the similarities and differences between these types of blood flow, providing valuable insights into the blood flow supply to working muscles.

The purpose of this study was to simultaneously measure femoral artery blood flow by pulsed-wave Doppler ultrasonography and intramuscular blood flow by power Doppler ultrasonography during intermittent submaximal isometric knee extension. Blood flow plays a key role in delivering oxygen to the working muscles. The demand for oxygen increases during muscle contraction; thus, increased blood flow provides the necessary oxygen. Given the relationship between intramuscular blood flow and muscle deoxygenation, comparing changes in them could provide further insights into muscle oxygen availability and utilization during exercise. Therefore, we also aimed to compare patterns of change in blood flow and muscle deoxygenation. We hypothesize that femoral artery blood flow would be proportional to exercise intensity (Osada et al. 2015), whereas intramuscular blood flow would plateau above moderate intensity (Izumi et al. 2025), and that the response of the femoral artery blood flow would not show a similar trend to that of intramuscular blood flow.

## Materials and methods

### Participants

Seventeen healthy young males participated in this study. This study was approved by the Ethics Committee of the Research Center of Health, Physical Fitness & Sports at Nagoya University (No. 21–05), and it was conducted in accordance with the Declaration of Helsinki. Before the experiments, the purpose, risks, and benefits of the study were explained to the participants, and written informed consent was obtained. All participants refrained from smoking, drinking caffeine and vigorous exercise at least 12 h before the experiments.

### Experimental procedures

All experiments were conducted in a laboratory under a controlled temperature of 22–24℃. Each participant came to the laboratory to familiarize themselves with the maximal voluntary contraction (MVC) test and the intermittent and submaximal isometric knee extension tasks. The experiment was performed at least 1 week after familiarization.

The participants performed an isometric knee extension MVC test using a custom-designed dynamometer (M-12297–3; Takei Scientific Instruments Co. Ltd., Niigata, Japan) with a mounted force transducer (LTZ-100 KA; Kyowa Electronic Instruments Co. Ltd., Tokyo, Japan). Throughout the knee extension tasks, the hip, chest, and ankle were fixed to the seat using straps, the hip joint angle was 70°, and the knee joint angle was 90° flexion. The participants were instructed to cross their arms in front of their chest. The MVC test comprised three phases: the force-rising phase (1–2 s), the sustained phase (≥ 2 s), and the relaxation phase (Watanabe and Akima 2009). The participants were actively encouraged by the supervisors to exert maximal effort. Three MVC tests were performed with ≥ 2-min rest between tests after the submaximal contractions for warm-up. The knee extension force, sampled at 400 Hz through an analog-to-digital converter (PowerLab 16SP; ADInstruments, Melbourne, Australia), was recorded and stored in a personal computer (Mac Mini; Apple Inc., Cupertino, CA, US). The peak value during each contraction was determined according to the MVC force. If the two highest exerted forces differed by 5% between trials, an additional trial was performed. The MVC force was determined as the highest force of all trials, and it was used to calculate the target force for the intermittent and submaximal contraction tasks.

After 10 min of rest following the MVC session, the intermittent (5 s on, 5 s off) isometric knee extension task was conducted at submaximal effort. Five contractions were performed at each force level (10%, 30%, 50%, and 70% of MVC) at random with a 10-min rest between sets. Each trial consisted of a 1-min baseline period before the exercise and a 2-min post-exercise phase. On the basis of visual feedback on the personal computer monitor with the target line and auditory feedback from the metronome, the participants completed the experiment.

### Femoral artery blood flow

Doppler ultrasonography (Vivid i; GE Healthcare Japan, Tokyo, Japan) was used to measure right femoral artery blood flow using an 8.8-MHz multifrequency linear probe. The femoral artery diameter was covered by the sample volume, and the pulsed-wave Doppler insonation angle was 60°. Images of the femoral artery and the relevant velocity wave from ultrasonography were visualized on a computer at a frequency of ~ 40 Hz using a capture device (DVI2USB 3.0; Epiphan Video, Ottawa, ON, Canada). The femoral artery diameter and mean blood velocity (MBV) were analyzed using custom-designed software (S-13037 v.2.5; Takei Scientific Instruments Co. Ltd.) (Katayama et al. 2019). Mean blood flow (MBF) in the femoral artery was calculated as follows: MBF = MBV × π × (diameter ÷ 2)^2^ × 60.

### Intramuscular blood flow

Power Doppler ultrasonography (LOGIQ e Premium; GE Healthcare, Wauwatosa, WI, US) equipped with a 12-MHz linear array probe (probe width, 3.8 cm) was used to measure intramuscular blood flow in the vastus lateralis during intermittent and submaximal muscle contraction, as previously described (Izumi et al. 2025). Using a custom-made styrene frame, the probe was placed perpendicular to the predicted longitudinal axis of the vastus lateralis on the skin. Transverse images were obtained at the mid-point between the lateral epicondyle and the greater trochanter of the femur of the right thigh. The probe was coated with sufficient transducer gel to provide acoustic contact without depression of the dermal surface. The power Doppler ultrasonography settings were held constant over all measurements with the following acquisition parameters: frequency, 6.3 MHz; gain, 30.5 dB; depth, 7 cm (Izumi et al. 2025). The Doppler window was set to show the whole image field. Power Doppler images obtained by ultrasonography were preserved on a personal computer (ENVY; Hewlett-Packard Japan, Tokyo, Japan) using a capture device (DVI2USB 3.0; Epiphan Video) in AVI format at a sampling rate of 20 frames per second.

Intramuscular blood flow was analyzed using custom-designed power Doppler signal-measuring software (S-22028 version 1.0.3; Takei Scientific Instruments Co. Ltd.) (Izumi et al. 2025). This software was developed to count the number of power Doppler signal pixels in the image. Intramuscular blood flow was assessed by the relative area of the power Doppler signal in the region of interest according to the following equation (Dori et al. 2016; Izumi et al. 2025): intramuscular blood flow (%) = (cross-sectional area of power Doppler signal) ÷ (cross-sectional area of region of interest) × 100.

Rectangular selections at the frame where no muscle contraction occurred and where there was no motion artifact during muscle movement were used to identify the region of interest in the vastus lateralis. As much muscle as feasible was included in the chosen location while avoiding obvious fascia.

### Muscle oxygenation

A spatially resolved near-infrared spectroscopy (NIRS) system (Hb14; Astem Co. Ltd., Kanagawa, Japan) with a dual-wavelength (770 and 830 nm) light-emitting diode was used to assess muscle oxygen saturation (StO_2_) in the vastus lateralis, as previously described (Akima and Ando 2017). The NIRS system consisted of a single computerized control segment and probe with 2.0 and 3.0 cm optode distances. Oxyhemoglobin + myoglobin, deoxyhemoglobin + myoglobin (deoxy-[Hb + Mb]), and total hemoglobin + myoglobin were determined by measuring the light attenuation at wavelengths of 770 and 830 nm. Light attenuation was analyzed using algorithms based on a modified Beer–Lambert law (Kime et al. 2010). The thickness of subcutaneous fat in the region at which the NIRS signal was recorded was measured using B-mode ultrasonography (LOGIQ e Premium; GE Healthcare). Subcutaneous fat thickness (i.e., the path length) was used to minimize errors by affecting scattering coefficient and to identify the relative change in Hb + Mb and the absolute StO_2_ values by NIRS application on a personal computer (ENVY; Hewlett-Packard Japan) (Niwayama et al. 2007). The relative absorption coefficients derived from the light attenuation slope over a distance measured at two focal sites from the light emission were used to determine the absolute StO_2_ values, which were determined by the NIRS system. The NIRS probe was placed perpendicular to the estimated longitudinal axis of the vastus lateralis at the distal neighbor to the ultrasound probe, which was used to determine intramuscular blood flow. To avoid interference by unwanted light, the probe was wrapped with double-sided adhesive tape and screened with elastic therapeutic tape. The NIRS data were transmitted to a personal computer (ENVY; Hewlett-Packard Japan) via a wireless connection (Bluetooth 2.0) after sampling at 2 Hz.

### Data analysis

The resting values of all parameters were averaged over 1 min just before the exercise protocol. The contraction and relaxation phases were distinguished by feeding the power Doppler video frame by frame. The femoral artery blood flow was averaged during both contraction and relaxation phases, and intramuscular blood flow was averaged during the relaxation phase. Femoral artery blood flow during the contraction phase was analyzed without a gap between contraction and relaxation, as previously reported (Osada et al. 2015). Femoral artery and intramuscular blood flow during the post-exercise phase were averaged every 5 s from the end of the fifth contraction, and the highest value during the post-exercise phase at each intensity was defined as the peak value. The total response was assessed as the area under the curve (AUC) from the end of exercise to 60 s post-exercise. The AUC was calculated using the caTools package in R-studio (http://www.rstudio.com). Both femoral artery and intramuscular blood flow were normalized to the peak value during the post-exercise phase within each exercise intensity for each participant and represented as %Peak. The normalization was conducted to compare patterns of change in variables with different units (femoral artery blood flow; ml/min, intramuscular blood flow; %). Time-to-peak was defined as the time from the end of exercise to the peak. StO_2_ and deoxy-[Hb + Mb] were represented as an absolute change (Δ) from baseline to minimums and maximums, respectively, during the post-exercise for each exercise intensity.

### Statistical analysis

All values are expressed as the mean ± standard deviation. The paired *t* test was used to compare femoral artery blood flow between contraction and relaxation phase, and StO_2_ and deoxy-[Hb + Mb] between baseline and post-exercise. One-way analysis of variance (ANOVA) with repeated measures was used to compare femoral artery blood flow, intramuscular blood flow, and StO_2_ across the exercise intensities. Two-way (measurement site × exercise intensity) ANOVA with repeated measures was used to identify differences in the %Peak and time-to-peak between femoral artery and intramuscular blood flow. When ANOVA showed a significant difference, Bonferroni’s post hoc test was performed to determine where the significant differences were found. Mauchly’s sphericity test was used to verify the sphericity assumption, and if violated, the Greenhouse–Geisser correction was applied. Statistical analyses were performed using IBM SPSS statistics software (version 27.0; IBM Corp., Tokyo, Japan). The level of significance was set at *P* < 0.05.

## Results

### Physical characteristics

The physical characteristics were as following: age, 19.7 ± 1.2 years; height, 170.7 ± 6.5 cm; weight, 58.5 ± 7.1 kg.

### Changes in femoral artery and intramuscular blood flow during intermittent isometric knee extension

**Fig. 1:**
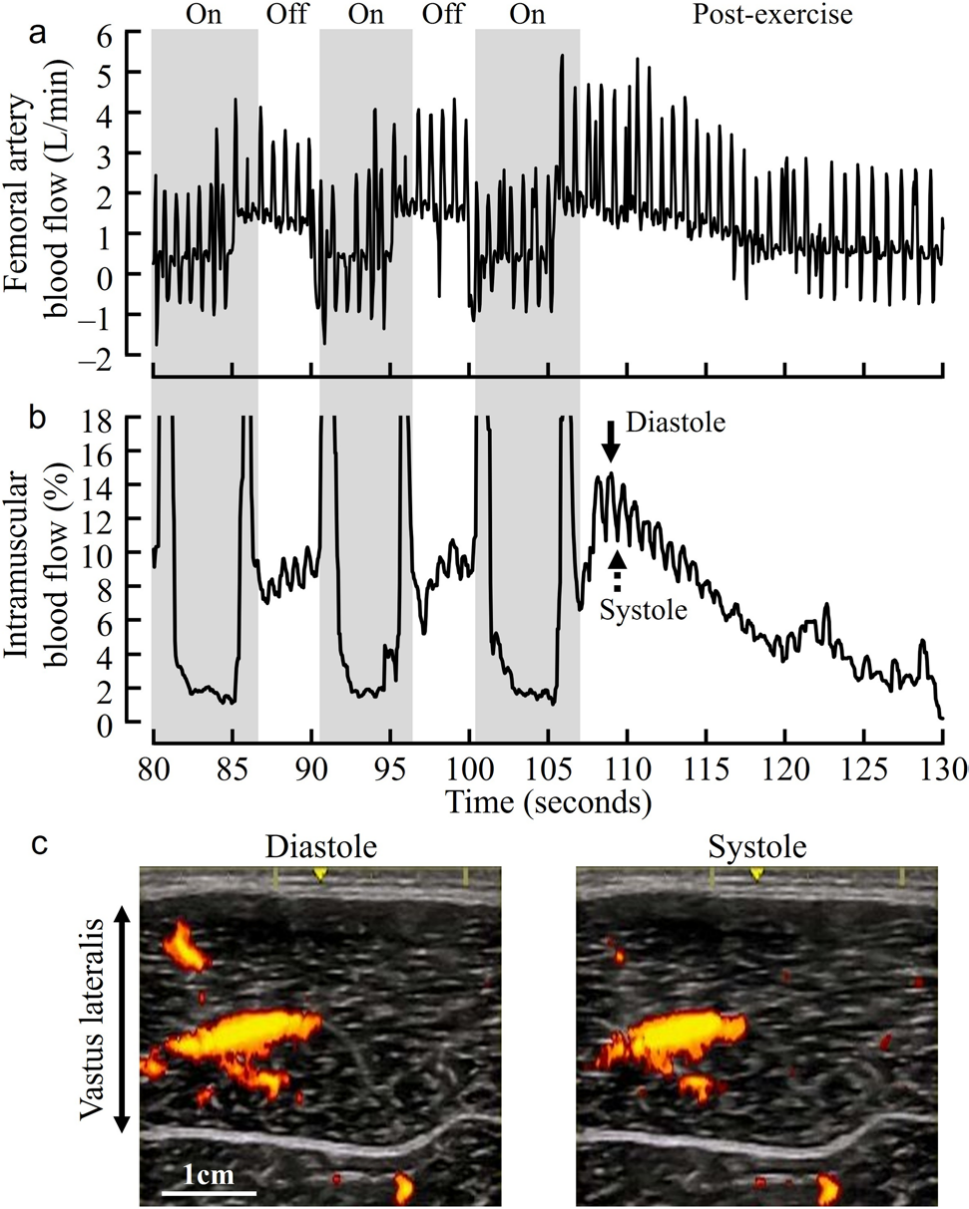
Representative recordings of femoral artery blood flow (**a**) and intramuscular blood flow (**b**) at 30% maximal voluntary contraction, and power Doppler images at diastole and systole during the post-exercise phase (**c**)

Figure 1 shows representative recordings of femoral artery blood flow (Fig. 1a) and intramuscular blood flow (Fig. 1b) at 30% MVC, and power Doppler images at diastole and systole during the post-exercise phase (Fig. 1c). Femoral artery and intramuscular blood flow during intermittent isometric knee extension are shown in Table 1. Compared to baseline, femoral artery blood flow during the muscle contraction phase was significantly greater only at 10% MVC (*P* < 0.01). No significant differences were observed at 30% (*P* = 0.58), 50% (*P* = 0.56), and 70% (*P* = 0.56) MVC. Femoral artery blood flow during the muscle relaxation phase was significantly higher than during the muscle contraction phase at all exercise intensities (all *P* < 0.01). Additionally, intramuscular blood flow was significantly higher than at baseline at all exercise intensities (all* P* < 0.01), and significantly higher than 10% MVC at 30% MVC (*P* < 0.01). Intramuscular blood flow could not be analyzed during the muscle contraction phase due to motion artifacts.

**Table 1:** Mean values for femoral artery and intramuscular blood flow during intermittent isometric knee extension exercises

Variables	Baseline	Contraction phase				Relaxation phase			
		10% MVC	30% MVC	50% MVC	70% MVC	10% MVC	30% MVC	50% MVC	70% MVC
Femoral artery blood flow (L/min)	0.29 ± 0.09	0.49 ± 0.17†	0.34 ± 0.13§	0.34 ± 0.17	0.34 ± 0.12	1.14 ± 0.37†‡	2.25 ± 0.74†‡¶	3.12 ± 0.88†‡¶	3.73 ± 0.99†‡§
Intramuscular blood flow (%)	0.09 ± 0.10	–	–	–	–	1.76 ± 1.76*	5.72 ± 4.19†¶	6.24 ± 3.82†	7.05 ± 4.53†

Values are means ± SD; *n* = 17. MVC, maximal voluntary contraction

^*^P < 0.05, †*P* < 0.01 vs. baseline

‡*P* < 0.01 contraction vs. relaxation

§*P* < 0.05

¶*P* < 0.01 vs. the one-lower %MVC

### Femoral artery and intramuscular blood flow during post-exercise

**Fig. 2:**
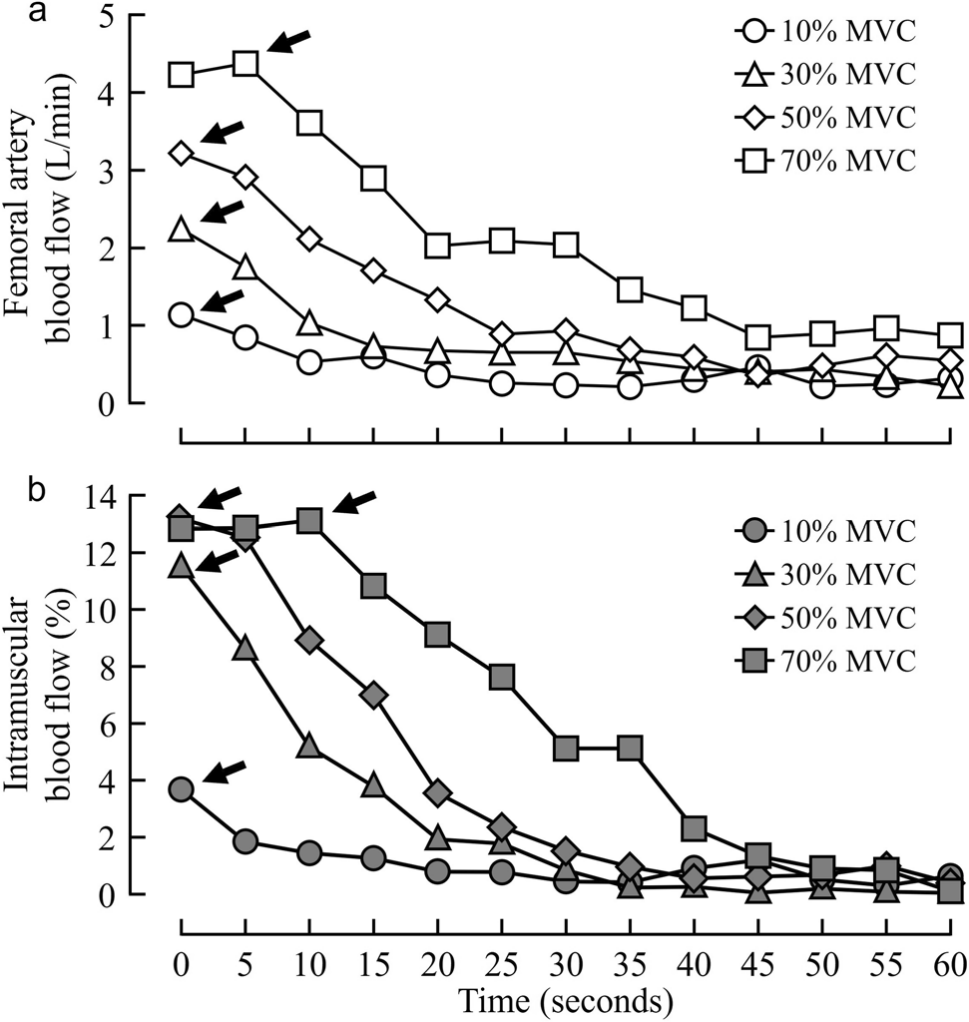
Representative changes in femoral artery blood flow (**a**) and intramuscular blood flow (**b**) on 5-s averages during the post-exercise phase. The arrows show the peak value at each exercise intensity. MVC, maximal voluntary contraction

**Fig. 3:**
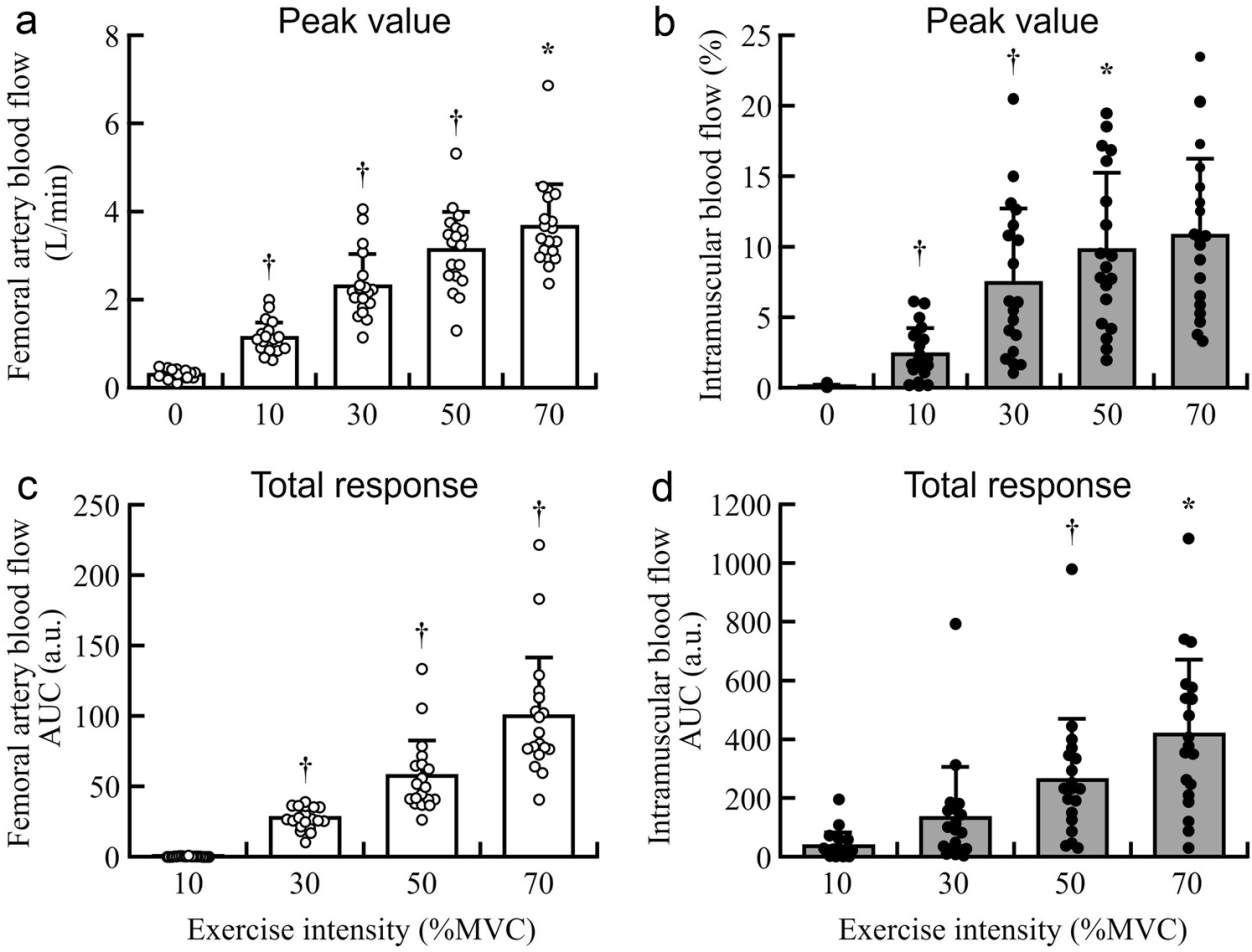
Individual and group-averaged values of the peak values (**a**, **b**) and total response (**c**, **d**) for femoral artery blood flow (white plots and bars) and intramuscular blood flow (gray plots and bars). *n* = 17. MVC, maximal voluntary contraction; AUC, area under the curve. a.u., arbitrary units. * *P* < 0.05, † *P* < 0.01 vs. the one-lower %MVC

Figure 2 shows representative changes in femoral artery (Fig. 2a) and intramuscular blood flow (Fig. 2b) on 5-s averages during the post-exercise phase. The arrows indicate the peak value at each exercise intensity. The peak values and total response (AUC) at each exercise intensity after exercise are illustrated in Fig. 3. Compared with the one-lower %MVC, the peak value during the post-exercise of femoral artery blood flow was significantly higher at 10% to 70% MVC (*P* < 0.01) (Fig. 3a). By contrast, the peak value during the post-exercise of intramuscular blood flow was significantly higher at 10% to 30% MVC (*P* < 0.01), and there was no significant difference between 30% and 50% MVC (*P* = 0.05) or between 50% and 70% MVC (*P* = 0.36), compared to the one-lower %MVC (Fig. 3b). The total response of femoral artery blood flow after the end of exercise was significantly higher with increasing exercise intensity (*P* < 0.01) (Fig. 3c). The total response of intramuscular blood flow during the post-exercise was significantly higher at 50% MVC and 70% MVC (*P* = 0.01) compared with the one-lower %MVC (Fig. 3d).

**Fig. 4:**
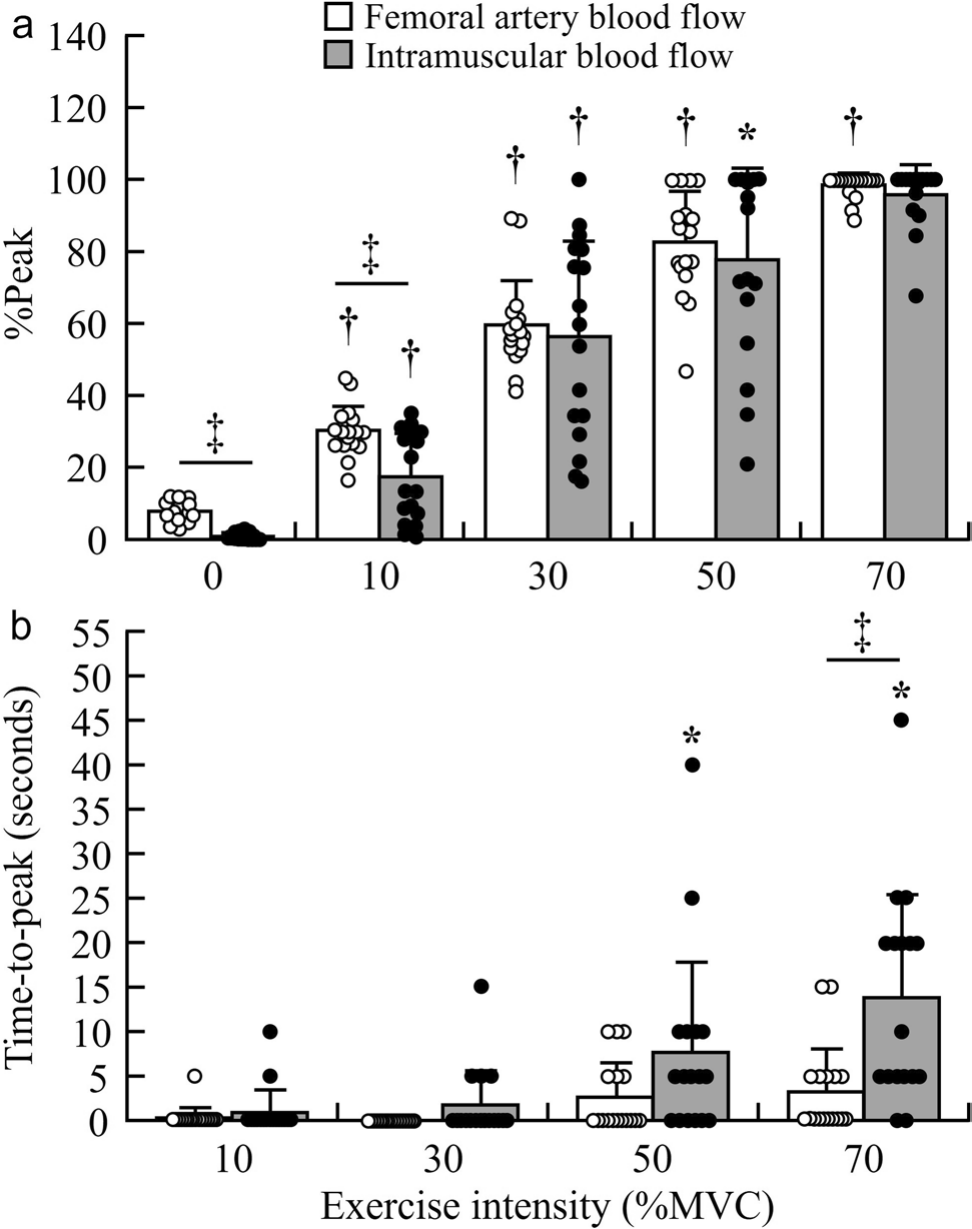
Individual and group-averaged values of the %Peak (**a**) and time-to-peak (**b**) for femoral artery blood flow (white plots and bars) and intramuscular blood flow (gray plots and bars) during the post-exercise phase. *n* = 17. MVC, maximal voluntary contraction; AUC, area under the curve. * *P* < 0.05, † *P* < 0.01 vs. the one-lower %MVC. ‡ *P* < 0.01 significant differences between femoral artery and intramuscular blood flow

The comparisons of the %Peak and time-to-peak between the femoral artery and intramuscular blood flow are depicted in Fig. 4. No significant interaction was observed between measurement site (femoral artery and vastus lateralis) and exercise intensity for the %Peak (*F* = 1.01, *P* = 0.37). However, a main effect of both measurement site and exercise intensity was observed (*F* = 5.97, *P* = 0.03 and *F* = 230.66, *P* < 0.01, respectively). The %Peak during the post-exercise of femoral artery blood flow was significantly higher than that of intramuscular blood flow at baseline (*P* < 0.01) and at 10% MVC (*P* < 0.01). The %Peak during the post-exercise of femoral artery blood flow significantly increased in proportion to exercise intensity (*P* = 0.01), and that of intramuscular blood flow significantly increased in an exercise intensity-dependent manner up to 50% MVC (*P* = 0.04) (Fig. 4a). Additionally, there was a significant interaction between measurement site (femoral artery and vastus lateralis) and exercise intensity for the time-to-peak (*F* = 8.04, *P* < 0.01), and a main effect of both measurement site and exercise intensity (*F* = 9.73, *P* = 0.01 and *F* = 16.98, *P* < 0.01, respectively) was observed. The time-to-peak of femoral artery blood flow was not significantly different between the exercise intensities (*P* = 0.09). The time-to-peak of 0 s means that the value immediately reached a peak after the exercise was completed, i.e. during the post-exercise phase. The time-to-peak during the post-exercise of intramuscular blood flow was significantly longer at 50% (*P* = 0.04) and 70% MVC (*P* < 0.05) compared with the one-lower %MVC. Moreover, the time-to-peak of intramuscular blood flow was significantly longer than that of femoral artery blood flow at 70% MVC (*P* < 0.01) (Fig. 4b).

### Muscle oxygenation after intermittent submaximal isometric knee extension

The StO_2_ and deoxy-[Hb + Mb] values are displayed in Table 2. A technical error resulted in the loss of NIRS data for two participants. Baseline StO_2_ was not significantly different among the exercise intensities (*P* = 1.00). In the baseline and post-exercise comparisons, StO_2_ at 10% MVC did not decrease significantly (*P* = 0.58), while StO_2_ at 30%, 50%, and 70% MVC decreased significantly with intermittent submaximal isometric knee extension (all *P* < 0.01). In the comparison among exercise intensities, ΔStO_2_ was significantly lower at 30% MVC (*P* = 0.03) and at 50% MVC (*P* < 0.01), but there was no significant difference between 50% and 70% MVC (*P* = 0.21) compared to the one-lower %MVC. Baseline deoxy-[Hb + Mb] was not significantly different among the exercise intensities (*P* = 1.00). Deoxy-[Hb + Mb] significantly increased from baseline to post-exercise at 30%, 50%, and 70% MVC (all *P* < 0.01), whereas there was no significant increase at 10% MVC (*P* = 0.42). A significant difference in Δdeoxy-[Hb + Mb] among the exercise intensities was observed between 10% and 30% MVC (*P* = 0.02) and between 30% and 50% MVC (*P* < 0.01).

**Table 2:** Mean values for muscle oxygenation

Variables		10% MVC	30% MVC	50% MVC	70% MVC
StO_2_ (%)	Baseline	69.51 ± 6.50	69.00 ± 5.80	69.83 ± 5.26	69.82 ± 7.27
	Post-exercise	70.29 ± 4.87	64.46 ± 5.21*	54.18 ± 10.47*	48.79 ± 10.16*
	Δ	0.78 ± 5.19	–4.54 ± 5.12†	–15.65 ± 9.14‡	–21.03 ± 7.73
Deoxy-[Hb + Mb] (M)	Baseline	19.33 ± 5.76	19.71 ± 4.61	19.14 ± 4.76	19.06 ± 5.96
	Post-exercise	20.03 ± 5.67	25.82 ± 6.88*	35.16 ± 11.69*	38.50 ± 12.17*
	Δ	0.71 ± 3.16	6.11 ± 4.89†	16.02 ± 8.86‡	19.44 ± 7.70

Values are means ± SD; *n* = 15. Δ shows absolute change from baseline to post-exercise. StO_2_, muscle oxygen saturation; Deoxy-[Hb + Mb], deoxy-hemoglobin + myoglobin; MVC, maximal voluntary contraction

*P < 0.01 vs. baseline at same exercise intensity

†*P* < 0.05

‡*P* < 0.01 vs. the one-lower %MVC in Δ

## Discussion

The purpose of this study was to evaluate the patterns of change in femoral artery and intramuscular blood flow during intermittent submaximal isometric knee extension. This was achieved using pulsed-wave Doppler ultrasonography for the measurement of femoral artery blood flow and power Doppler ultrasonography for the evaluation of intramuscular blood flow, simultaneously. A significant interaction between femoral artery and intramuscular blood flow was observed for the time-to-peak (Fig. 4b), but not for the %Peak (Fig. 4a). These results indicate that the patterns of change in femoral artery and intramuscular blood flow were not different during the post-exercise phase. However, the time-to-peak of intramuscular blood flow was slower than that of femoral artery blood flow. These results suggest that differences in vascular reactivity and blood flow regulation exist between the femoral artery and skeletal muscle microvasculature.

### Changes in femoral artery and intramuscular blood flow during intermittent isometric knee extension

Owing to elevated intramuscular pressure and mechanical compression of the blood vessels during the contraction phase, most of the oxygen is supplied during the relaxation phase of the contraction–relaxation cycle of intermittent contraction (Broxterman et al. 2014; Osada et al. 2015; Hammer et al. 2024). In the present study, femoral artery blood flow during the muscle contraction phase significantly increased at 10% MVC but not at 30% MVC or higher, compared to baseline. This result is consistent with a previous study by Richardson (1981), who showed that calf muscle blood flow during static contraction below 15% MVC was higher than the resting value. Because of motion artifacts, the changes in intramuscular blood flow during muscle contraction remain unknown.

Intramuscular blood flow significantly increased at 30% MVC during the muscle relaxation phase, but significant increases were not observed above 30% MVC compared with the one-lower exercise intensity. According to a previous study, post-contraction hyperemia is inversely related to blood flow restriction during contraction (Wigmore et al. 2006). However, the absence of an increase in intramuscular blood flow at 30% MVC or higher may be due to the onset of the subsequent contraction initiated prior to the intramuscular blood flow reaching a sufficient level during the muscle relaxation phase.

### Differences between femoral artery and intramuscular blood flow during post-exercise

We hypothesized that the patterns of change in femoral artery and intramuscular blood flow would differ during intermittent isometric knee extension at submaximal force exertion. There was no significant interaction between measurement site (femoral artery and vastus lateralis) and exercise intensity for the %Peak, representing that the pattern of change was similar between femoral artery and intramuscular blood flow. However, the main effects of measurement site and exercise intensity were observed. The %Peak of femoral artery blood flow was significantly greater at higher exercise intensities (Fig. 4a), and the peak value was also significantly higher at higher exercise intensities (Fig. 3a). Conduit artery blood flow after exercise increases to meet the oxygen demand as exercise intensity rises, and the results of the present study are consistent with those of previous studies (Hunter et al. 2009; Osada et al. 2015). At lower exercise intensities, both the %Peak and peak values of intramuscular blood flow were significantly higher with higher exercise intensities (Fig. 3b, 4a). However, the values were not significantly higher at higher force levels. Blood flow is influenced by changes in blood pressure. Previous studies have reported that arterial blood pressure was greater at the end of higher intensity exercise (Lutjemeier et al. 2005; Hammer et al. 2021). In addition, higher exercise intensity was reported to have higher leg vascular conductance during the recovery period (Hammer et al. 2021). This increase in driving pressure due to high arterial blood pressure and elevated vascular conductance may have resulted in higher blood flow at higher exercise intensities. Thus, it is necessary to consider the possibility that the changes in blood flow recovery may be related to differences in blood pressure changes with exercise intensity. However, blood pressure was not measured; unfortunately, we were unable to determine its impact on the observed blood flow responses in the present study. The difference in the hemodynamic pattern between the femoral artery and skeletal muscle microvasculature may reflect heterogeneity in the blood flow responses of the quadriceps muscle. Heterogeneity is known as the unequal distribution of blood flow among muscles (Heinonen et al. 2015). Although the blood flow distribution has demonstrated heterogeneity in the quadriceps femoris (Kalliokoski et al. 2000; Heinonen et al. 2007), intramuscular blood flow has demonstrated homogeneity with increasing exercise intensity due to blood flow re-distribution to the newly recruited motor units (Heinonen et al. 2007; Hammer et al. 2018). Deoxy-[Hb + Mb] responses are associated with the differences in the muscle recruitment patterns among individual quadriceps muscles during incremental cycling exercise (Chin et al. 2011). In our previous study, the relationship between the normalized electromyographic activity level and normalized force during isometric knee extension was found to be non-linear in all four quadriceps muscles, including the vastus intermedius (Watanabe and Akima 2009). Additionally, neuromuscular activation in the rectus femoris was elevated at higher intensities (Watanabe and Akima 2009). Therefore, the blood flow in the vastus lateralis may not have differed significantly between 50% and 70% MVC because more femoral artery blood flow was distributed to the rectus femoris, where neuromuscular activity would have been greater during muscle contraction at high intensity (Watanabe and Akima 2009).

The time-to-peak was slower for intramuscular blood flow than for femoral artery blood flow following contractions at 70% MVC (Fig. 4b). Conduit arteries, such as the femoral artery, are positioned in serial pathways with skeletal muscle, whereas skeletal muscle micro vessels have a branched parallel structure (Poole et al. 2013). The positional relationship of these vessels is thought to influence differences in hemodynamics during exercise. A delay in the increase in intramuscular blood flow relative to that of the conduit artery has been also reported by Harper et al. (2006) and Ichinose et al. (2021). However, in the study by Ichinose et al., the time-to-peak was consistently approximately 5 s slower in the skeletal muscle microvasculature than in the brachial artery at 20%, 40%, 60%, and 80% MVC (Ichinose et al. 2021). The study compared temporal changes in blood flow between the brachial artery and skeletal muscle microvasculature of the flexor digitorum during 1-s handgrip exercise. We speculate that this discrepancy between the current and previous study could be due to differences in the methodology, measurement sites, and/or exercise protocols used. Alternatively, the differences may also be attributed to limb-specific differences in blood flow regulation mechanisms between the upper and lower limbs (Donato et al. 2006).

Intramuscular pressure increases with isometric contraction at 30% MVC or higher, and this higher intramuscular pressure could strongly compress the microvasculature (Sadamoto et al. 1983). Clifford et al. (2006) have reported that increased extravascular pressure activates an intrinsic mechanosensitive mechanism that causes vasodilation in rat soleus muscle, so the vasodilation may have been stronger or longer at higher intensities. Ichinose et al. (2021) have reported delayed time-to-peak of forearm skeletal muscle microvasculature after handgrip exercise above 60% MVC, which is consistent with the results of this study. Furthermore, when muscle contraction intensity is low and femoral artery blood flow is less restricted, blood may be allowed to flow into the skeletal muscle microvasculature during the contraction phase, and thus there is no significant increase in blood flow during the relaxation phase. On the other hand, when femoral artery blood flow is restricted, most of the blood flow to the muscle is supplied during the relaxation phase, and the degree of blood flow increase may be greater. Therefore, it is anticipated that the exercise intensity at which the time-to-peak would be delayed correlate with that of femoral artery blood flow restriction. However, the time-to-peak of intramuscular blood flow after contractions was slower above 50% MVC, which did not correspond to the intensity of femoral artery blood flow restriction (above 30% MVC). Unfortunately, the reason for this disagreement was not well understood. The difference between the peak value and the total response may be related to the differences observed. In addition, the time-to-peak of intramuscular blood flow may be influenced by local vasodilation (Tschakovsky et al. 2004) and not be explained solely by the blood flow limiting intensity of the femoral artery.

### Response of muscle oxygenation to intermittent submaximal knee extension

StO_2_ reflects the balance between oxygen supply and consumption in the muscle, and a decrease in StO_2_ indicates that the oxygen consumption exceeds the oxygen supply (McCully and Hamaoka 2000). An elevation in deoxy-[Hb + Mb] reflects an increase in fractional oxygen extraction, which is influenced by both muscle metabolism and blood flow (DeLorey et al. 2003). Thus, an increase in deoxy-[Hb + Mb] may result from either an increase in oxygen consumption or a decrease in oxygen delivery, or both. Although StO_2_ significantly decreased and deoxy-[Hb + Mb] significantly increased post-exercise above 30% MVC compared with baseline, there were no significant differences in these values between baseline and post-exercise at 10% MVC (Table 2). Blood flow plays a key role in delivering oxygen to the working muscles. The demand for oxygen increases during muscle contraction; thus, increased blood flow provides the necessary oxygen. However, elevated mechanical compression due to muscle contraction obstructs arterial inflow (Damon et al. 2007). The present study demonstrated that femoral artery blood flow during isometric muscle contraction was not inhibited at 10% MVC; however, it was occluded at a level equivalent to the resting state at 30% MVC and above (Table 1). It is thus considered that StO_2_ and deoxy-[Hb + Mb] did not change from baseline to the end of the intermittent knee extension task (i.e., there was a balance between oxygen demand and blood flow supply), because femoral artery blood flow significantly increased from baseline to contraction phase at 10% MVC, and oxygen was supplied during muscle contraction at 10% MVC. The femoral artery blood flow during 10% MVC was sufficient to meet the increased oxygen demand, as indicated by the unchanged StO_2_ and deoxy-[Hb + Mb] values. In contrast, at 30% MVC and above, the increase in oxygen consumption and the reduction in oxygen delivery due to mechanical compression restricted blood flow, and the balance between oxygen supply and demand would be disrupted, leading to a significant increase in deoxy-[Hb + Mb] (Table 2). These findings indicate that exercise-induced deoxygenation is influenced by both the mechanical restriction of blood flow and the increased oxygen consumption associated with higher contraction intensities.

## Limitations and experimental considerations

Heterogeneity in blood flow within and among the working muscles may be associated with differences in muscle fiber types and muscle activity levels (Kalliokoski et al. 2000; Laaksonen et al. 2006; Heinonen et al. 2007). The present study assessed intramuscular blood flow at the vastus lateralis of the mid-thigh. Intramuscular blood flow changes may depend on the measurement site; therefore, it is necessary to assess intramuscular blood flow at different sites of the vastus lateralis, such as distal and/or proximal regions, as well as at other synergistic muscles, such as the rectus femoris or vastus medialis. In addition, we evaluated intramuscular blood flow based on the relative area of power Doppler signals within the region of interest, which mainly indicates blood flow. Therefore, it should be noted that intramuscular blood flow is not expressed as an absolute value; changes in intramuscular blood flow could only be interpreted relative to baseline. Thus, the discrepancy between the units of femoral artery blood flow (L/min) and intramuscular blood flow (%) prevents a direct comparison of their patterns of change. We recruited only young male individuals in this study. The measured parameters may be affected by the effects of sex and age on alterations in muscle perfusion both during and after exercise (Rudroff et al. 2014; Hunter 2014). Therefore, future studies involving female individuals and elderly people are needed to examine differences in the response to exercise by sex and age. Finally, it should be noted that the measurement regions of intramuscular blood flow and muscle deoxygenation were not completely the same. Intramuscular blood flow was analyzed to the depth of the vastus lateralis, whereas the variables of muscle deoxygenation are thought to be measured within the superficial muscle.

## Conclusions

We compared the changing patterns of femoral artery and intramuscular blood flow during intermittent submaximal knee extension. The patterns of change in femoral artery and intramuscular blood flow were not different in quantity; however, the time-to-peak of intramuscular blood flow was slower than that of femoral artery blood flow during the post-exercise phase. These results indicate that the changes in pattern of femoral artery blood flow were different from those of intramuscular blood flow. This suggests that vascular reactivity and blood flow regulation differ between the femoral artery and skeletal muscle microvasculature during intermittent isometric knee extension at submaximal exertion in healthy young males.

## Data Availability

The datasets generated and analyzed during the current study are available from the corresponding authors upon reasonable request.

## Abbreviations

ANOVA: Analysis of variance
a.u.: Arbitrary units
AUC: Area under the curve
Hb + Mb: Hemoglobin + myoglobin
MBF: Mean blood flow
MVC: Maximal voluntary contraction
NIRS: Near-infrared spectroscopy
StO_2_: Muscle oxygen saturation
